# Secoisolariciresinol diglucoside is a blood-brain barrier protective and anti-inflammatory agent: implications for neuroinflammation

**DOI:** 10.1186/s12974-018-1065-0

**Published:** 2018-01-27

**Authors:** Slava Rom, Viviana Zuluaga-Ramirez, Nancy L. Reichenbach, Michelle A. Erickson, Malika Winfield, Sachin Gajghate, Melpo Christofidou-Solomidou, Kelly L. Jordan-Sciutto, Yuri Persidsky

**Affiliations:** 1 Department of Pathology and Laboratory Medicine, Temple University, Philadelphia, PA 19140 USA; 2 Center for Substance Abuse Research, Lewis Katz School of Medicine, Temple University, Philadelphia, PA 19140 USA; 3 Department of Pathology, School of Dental Medicine, University of Pennsylvania, Philadelphia, PA 19104 USA; 4 Department of Medicine, Perelman School of Medicine, University of Pennsylvania, Philadelphia, PA 19104 USA

## Abstract

**Background:**

Secoisolariciresinol diglucoside (SDG), the main lignan in flaxseed, is known for its beneficial effects in inflammation, oxidative stress, heart disease, tumor progression, atherosclerosis, and diabetes. SDG might be an attractive natural compound that protects against neuroinflammation. Yet, there are no comprehensive studies to date investigating the effects of SDG on brain endothelium using relevant in vivo and in vitro models.

**Methods:**

We evaluated the effects of orally administered SDG on neuroinflammatory responses using in vivo imaging of the brain microvasculature during systemic inflammation and aseptic encephalitis. In parallel, the anti-inflammatory actions of SDG on brain endothelium and monocytes were evaluated in vitro blood-brain barrier (BBB) model. Multiple group comparisons were performed by one-way analysis of variance with Dunnet’s post hoc tests.

**Results:**

We found that SDG diminished leukocyte adhesion to and migration across the BBB in vivo in the setting of aseptic encephalitis (intracerebral TNFα injection) and prevented enhanced BBB permeability during systemic inflammatory response (LPS injection). In vitro SDG pretreatment of primary human brain microvascular endothelial cells (BMVEC) or human monocytes diminished adhesion and migration of monocytes across brain endothelial monolayers in conditions mimicking CNS inflammatory responses. Consistent with our in vivo observations, SDG decreased expression of the adhesion molecule, VCAM1, induced by TNFα, or IL-1β in BMVEC. SDG diminished expression of the active form of VLA-4 integrin (promoting leukocyte adhesion and migration) and prevented the cytoskeleton changes in primary human monocytes activated by relevant inflammatory stimuli.

**Conclusion:**

This study indicates that SDG directly inhibits BBB interactions with inflammatory cells and reduces the inflammatory state of leukocytes. Though more work is needed to determine the mechanism by which SDG mediates these effects, the ability of SDG to exert a multi-functional response reducing oxidative stress, inflammation, and BBB permeability makes it an exciting potential therapeutic for neuroinflammatory diseases. SDG can serve as an anti-inflammatory and barrier-protective agent in neuroinflammation.

## Background

Secoisolariciresinol diglucoside (SDG), is the main lignan in wholegrain flaxseed, known for its beneficial effects including anti-inflammatory, antioxidant, anti-mutagenic, anti-microbial, anti-obesity, hypolipidemic, and neuroprotective effects. SDG ameliorates different types of diseases (cardiovascular, diabetes, lupus nephritis, menopause, reproduction, mental stress, immunity, atherosclerosis, hematopoietic, liver necrosis, and urinary disorders) as described in a recent review [1].

SDG has been shown to be effective in several pre-clinical models of diseases in which oxidative stress and inflammation play a prominent role in pathogenesis, including heart disease [2–4] and diabetes [5, 6]. In addition, SDG showed positive effects in cancer [4, 7, 8], liver [9, 10], and kidney inflammation as well as in obesity and the metabolic syndrome [10, 11]. Importantly, diverse SDG formulations have also been safely used in human clinical trials [4, 10, 12, 13].

The exact mechanisms of action of SDG are not fully elucidated. Known protective mechanisms include direct free radical scavenging activity [14–17], with significantly more efficiency than ascorbic acid (AA) and α tocopherol at reducing free radicals. Additional antioxidant activity in a cellular context could be attributed to activation of the endogenous antioxidant response (EAR), which mediates SDG mitigation of radiation-induced tissue damage [18]. Transcriptomics of SDG-treated alveolar macrophages indicate SDG as a potent EAR inducer and a repressor of inflammatory mediators such as cytokines and chemokines (i.e., TNFα, IL-5, IL-6, and IL-12) [18]. Further, SDG can attenuate respiratory bursts in activated lung macrophages [19] suggesting anti-inflammatory roles for SDG in this cell population.

In the current study, we investigated whether SDG oral administration will diminish neuroinflammation and BBB injury in relevant animal models including aseptic encephalitis induced by intracranial (i.c.) injection of TNFα. Indeed, oral administration of SDG attenuated leukocyte adhesion and migration across the BBB. Further, using in vitro BBB models, we replicated diminished inflammatory responses in brain endothelial cells and primary human monocytes and found potential mechanisms behind such effects.

## Methods

### Reagents

Reagents used in this study and their sources are as follows: Secoisolariciresinol diglucose (SDG) was chemically synthesized as a mixture of (S,S)- and (R,R)-isomers by Chemveda Life Sciences (San Diego, CA) and was reconstituted in sterile RNase, DNase-free water (Life Technologies, Carlsbad, CA) for in vitro experiments or in sterile saline (Life Technologies) for in vivo experiments. Recombinant human tumor necrosis factor alpha (TNFα) and human monocyte chemotactic protein type 1 (hMCP-1/CCL2) were from R&D Systems (Minneapolis, MN). Recombinant human interleukin beta (IL1β) was from PeproTech (Rocky Hill, NJ). Lipopolysaccharide (LPS) from *Escherichia coli* 0111:B4 was from Sigma-Aldrich (St. Louis, MO). The VLA-4-specific ligand, LDV (L-leucyl-L-aspartyl-L-valyl-L-prolyl-L-alanyl-L-alanyl-L-lysine) was from Bio-Techne (Minneapolis, MN). Rhodamine 6G was from Sigma-Aldrich (Saint Louis, MO).

### Cells

Isolation of primary human brain microvascular endothelial cells (BMVEC) was accomplished as previously described from normal brain resection tissue following surgery for treatment of intractable epilepsy [20] and provided by Michael J. Bernas and Dr. Marlys H. Witte (University of Arizona, Tucson, AZ). BMVEC were characterized and maintained as previously described from our laboratory [21]. Primary human monocytes were isolated and purified from the University of Nebraska Medical Center (Department of Pharmacology and Experimental Neuroscience, Omaha, NE) by counter current centrifugal elutriation as described [22] from HIV-1 and hepatitis B seronegative donors. BMVEC or monocytes were treated with different concentrations of SDG (0, 1, 2, 5, 10, or 50 μM) in accordance with previous studies [7, 17, 23].

### Adhesion assays

Monocytes were used within 24 h of isolation in adhesion assays following 16-h treatment with TNFα (20 ng/ml) in the absence or presence of SDG. All treatments were removed prior to labeling of the monocytes with calcein-AM (Life Technologies) as described [24]. Calcein-labeled monocytes were added to BMVEC monolayers stimulated by 16-h treatment with TNFα (20 ng/ml). Adhesion of monocytes to BMVEC was measured by fluorescence using a Synergy 2 plate reader (Biotek Instruments, Winooski, VT) from triplicate determinations and is expressed as fold difference in monocyte attachment (mean ± SEM) compared to baseline (untreated control).

### Migration assays

An in vitro model of BBB transendothelial migration was used to measure monocyte migration through BMVEC monolayers as previously described [24]. Monocytes and BMVEC were treated with TNFα and/or SDG as described above. Human recombinant MCP-1 (CCL2, 30 ng/ml) was added to the lower chamber of the BBB construct to promote migration of monocytes through the BMVEC monolayer. Migration was quantitated with ImageJ software (NIH, Bethesda, MD) from triplicate determinations and is expressed as fold difference in monocyte migration (mean ± SEM) compared to baseline (untreated control in the absence of MCP-1).

### Flow cytometry

Surface expression of vascular cell adhesion molecule 1 (VCAM-1) was measured by flow cytometry (FACS) as described [21]. BMVEC were pretreated in the absence or presence of SDG for 1 h followed by treatment with TNFα or IL1β (100 ng/ml) for 4 h. FITC-conjugated antibody to VCAM-1 (CD106) was from BD Biosciences (San Jose, CA). Actin cytoskeleton rearrangements were measured in monocytes treated in the absence or presence of the very late antigen 4 (VLA-4)-specific ligand, LDV peptide (12 nM), and SDG (10 or 50 μM, 4 h) as described [25]. HUTS-21 antibody, specific for the activated conformation of VLA-4, was detected by HUTS21 (R&D Systems), was measured by FACS as described [25, 26]. LFA-1 conformation, detected by MEM-148 Ab, was measured as described [27]. Quantitation of integrin conformational activation was performed where the mean fluorescence intensity (MFI) of activated non-treated cells was assigned a value of 100 and a value of 0 was assigned to the MFI of cell autofluorescence [25, 28, 29]. All other calculations were done utilizing the regression curve calculation tool of Prism v5 software (GraphPad Software Inc., La Jolla, CA). Fibrillar (F) and globular (G) forms of actin were quantitated with Acti-Stain Alexa-488 (Cytoskeleton Inc., Denver, CO) and DNase-1-Alexa 594 (Life Sciences), respectively. The F/G actin ratio was calculated by dividing mean fluorescence intensity (MFI) of F-actin by MFI of G-actin and the levels of F/G actin in non-stimulated, non-treated cells were assigned a value of 1. A FACS Canto II flow cytometer and FlowJo software version 8.7 (Tree Star, Ashland, OR) were used to acquire and analyze data as described [25, 29]. Data from at least 10,000 recorded events per treatment condition are presented as MFI (mean ± SEM from at least three experiments).

### Experimental animals

All in vivo experiments were approved by the Temple University Institutional Animal Care and Use Committee in accordance with guidelines based on the National Institutes of Health (NIH) guide for care and use of laboratory animals and ARRIVE (Animal Research: Reporting In Vivo Experiments) guidelines (www.nc3rs.org.uk/arrive-guidelines). SDG was administered to mice by oral administration using a non-forceful feeding technique, which consisted of hand feeding mice (10 weeks old, male) that had been previously trained to accept up to 30 μl volume of treatment solution. SDG doses were tailored to individual mouse weights and were freshly prepared 10 min before being offered to the mice. Mice were pretreated with SDG (4 mg/mouse) 2 h before i.c. administration of TNFα (0.5 μg/mouse) as an inflammatory insult. Administration of SDG formulations delivering a daily dose of 4 mg SDG/mouse (200 mg/kg) has been found to be protective in murine models of asbestos-induced inflammation [7] as well as radiation-induced inflammation [18, 30].

### Leukocyte adhesion to the BBB

Intravital video microscopy (IVM) (via cranial window) was used to quantify in vivo leukocyte adhesion in the presence or absence of SDG treatment and inflammatory insult [31]. 5 days after implantation of the cranial window with adjacent cannula [31], mice were injected with rhodamine 6G (0.1%). Serial images were obtained through the cranial window 2 h after inflammatory insult using a Stereo Discovery V20 epifluorescence microscope (Carl Zeiss Microimaging, Thornwood, NY) as described [31]. The number of adherent leukocytes was quantitated as previously described [31–33].

### FIn vivo permeability assay

Animals were injected i.p. injection of 200 μl of 2% sodium-fluorescein (Na-F) in saline. The amount of Na-F evaluated as described [21] was measured using a Synergy 2 plate reader (BioTek). Fluorescent dye content was calculated using external standards, and the data are expressed as amount of tracer per mg of tissue.

### Statistical analysis

Results are expressed as the mean ± SEM of experiments conducted numerous times. Multiple group comparisons were performed by one-way analysis of variance with Dunnet’s post hoc tests. Statistical analyses were performed utilizing Prism v6.0c software (GraphPad Software Inc., La Jolla, CA). Differences were considered significant at *p* values < 0.05.

## Results

### SDG administration diminishes leukocyte adhesion and migration across BBB in neuroinflammation

**Fig. 1:**
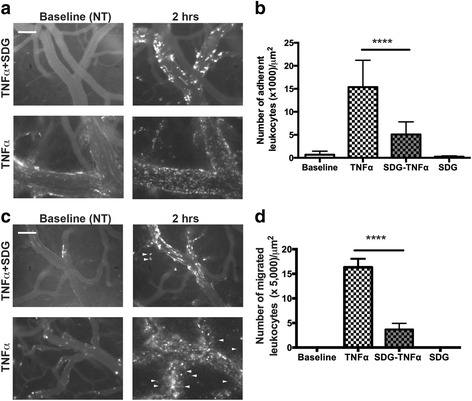
Orally administered SDG decreased leukocyte adhesion to and migration across the BBB. Representative images from videos of leukocytes labeled with 0.01% Rhodamine 6G are shown. 5 days after implanting cranial window with adjacent cannula, each mouse was pretreated with SDG at 4 mg/mouse, 2 h prior to TNFα injection i.c. (0.5 μg/mouse). Adherent and migrated leukocytes in animals with or without SDG treatment are shown in panels **a** and **c**, respectively. Quantitative analysis of leukocyte adhesion (**b**) to and migration (**d**) across the endothelium. Arrows point to migrated leukocytes. Experiments were performed on 5–8 mice in each group. ****p < 0.0001. Scale bar 100 μm is included in the upper left corner of one of the micrographs in **a** and **c**

It was documented before that SDG administration in vivo diminished end-organ injury in models of systemic inflammation and lung radiation injury. However, to date, there have not been studies assessing SDG effects on BBB in the setting of systemic inflammation or neuroinflammation. To address this question, we used a novel in vivo imaging technique, intravital microscopy through a cranial window, in our model of aseptic encephalitis following i.c. injection of TNFα, a cytokine overexpressed in many neuro-inflammatory conditions including multiple sclerosis, encephalitis, stroke, and HIV encephalitis [34–36]). Our prior work indicated that this stimulus enhances both leukocyte adhesion to and migration across the BBB [19]. Indeed, i.c. administration of TNFα led to a 20-fold increase in leukocyte adhesion and enhanced migration of leukocytes across the barrier 2 h later (Fig. 1), consistent with previous reports. Oral administration of SDG attenuated adhesion of leukocytes to the endothelium by 50% and attenuated migration of leukocytes across the BBB by 64% (Fig. 1). Leukocyte adhesion resolved 24 h later and SDG feeding therefore did not show a difference compared with the vehicle-treated control group after 24 h. Of note, administration of SDG alone did not affect leukocyte adhesion or migration.

**Fig. 2:**
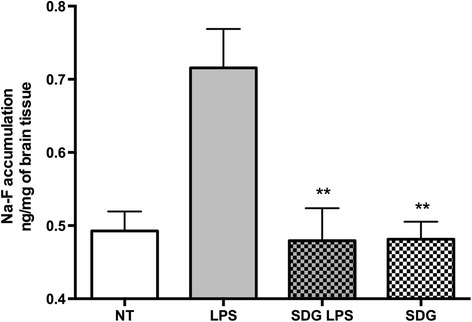
SDG diminishes BBB permeability in vivo. Quantification of Na-F accumulation in the brain in LPS-associated encephalitis. Experiments performed in triplicate with 5–7 mice in each group. The results are shown as mean adhesion ± SEM. **p < 0.01 is considered significant vs. LPS-treated

The systemic inflammatory response accompanied by leukocyte adhesion to brain endothelium results in BBB leakage [21]. Thus, we assessed BBB permeability in vivo using the low molecular weight tracer, sodium fluorescein (Na-F), after 4 h of LPS administration (Fig. 2). LPS administration led to increased BBB permeability (20%) that was prevented by SDG, pointing to barrier protective effects of SDG in vivo.

### SDG treatment attenuates human monocyte adhesion to and migration across human brain endothelial monolayers

**Fig. 3:**
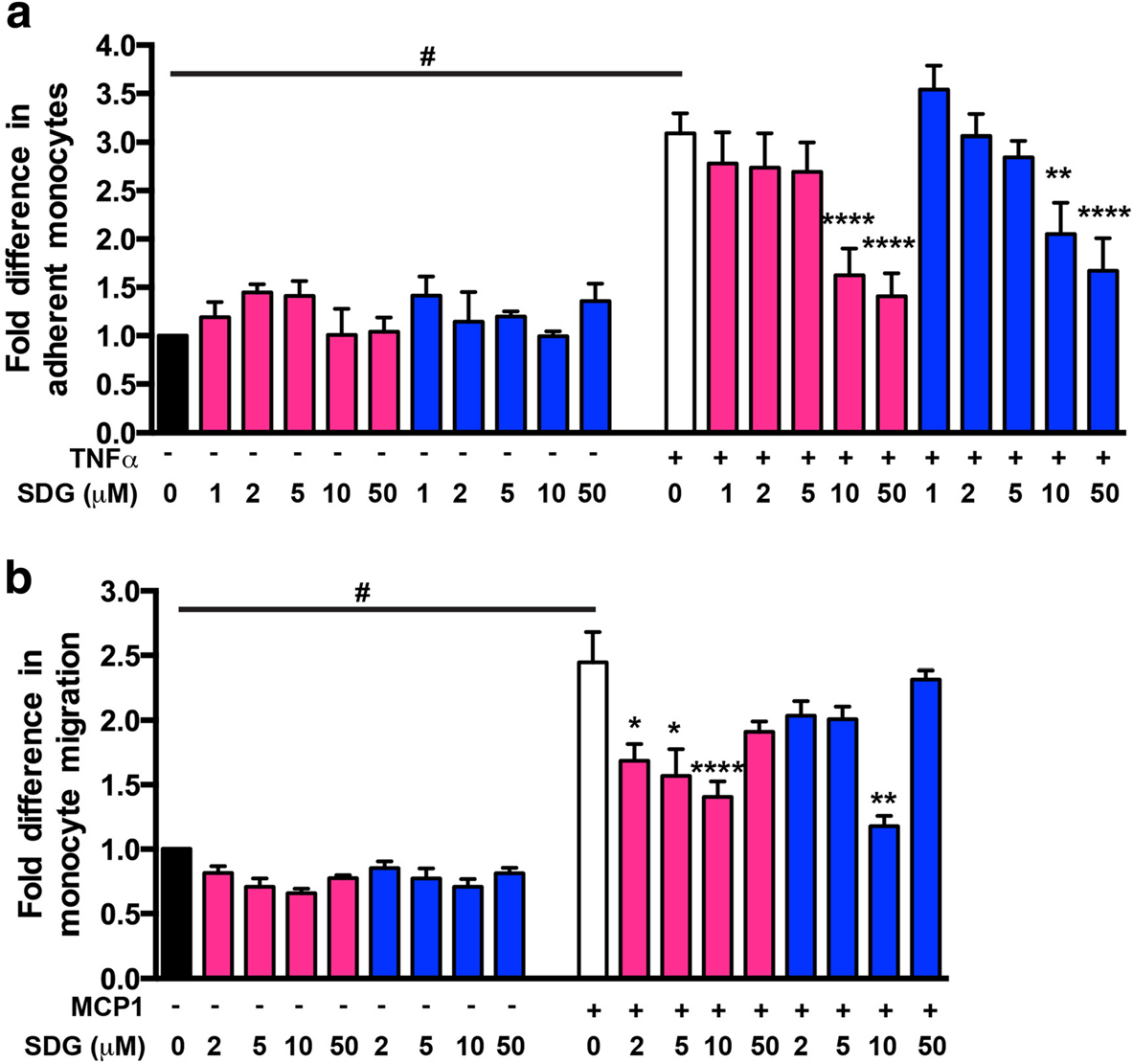
SDG treatment attenuates human monocyte adhesion to and migration across monolayers of human brain BMVEC. SDG diminished adhesion of TNFα-activated (20 ng/ml) human brain endothelial cells. BMVEC (red bars) or monocytes (blue bars) were treated for 24 h with SDG at 1, 2, 5, 10, or 50 μM prior to adhesion (panel **a**) or migration (panel **b**) assay. Means of quadruplicate determinations are shown ± SEM. #*p* < 0.0001 is considered significant vs. non-activated and non-treated cells. *p < 0.05, **p < 0.001, and ****p < 0.0001 is considered significant vs. TNFα-activated and non-treated human brain endothelial cells

In order to test whether SDG can diminish neuroinflammation in human tissues, we modeled inflammatory responses using monolayers of primary human brain endothelial cells (BMVEC). We tested whether SDG could decrease adhesion of primary human monocytes to TNFα-stimulated brain endothelium. BMVEC monolayers were activated by TNFα in the presence of SDG. Primary human monocytes were placed on the BMVEC only after all treatments were removed and the medium changed. TNFα upregulated monocyte adhesion 2.3-fold and SDG treatment of BMVEC (10 or 50 μM) diminished immune cell adhesion by 87% (Fig. 3a). Alternatively, we treated primary human monocytes with SDG and showed a decrease in adhesion of 54 or 79% after treatment with 10 or 50 μM SDG, respectively. Using migration assays in an in vitro BBB model, we tested whether SDG treatment of endothelial cells could prevent monocyte passage across BMVEC monolayers using CCL2 as a relevant cytokine. Application of CCL2 to the lower chamber of BBB constructs increased monocyte migration 2.6-fold. Pre-treatment of BMVEC with SDG attenuated monocyte migration across endothelial monolayers by 30–46% (Fig. 3b). Next, we studied whether pretreatment of monocytes with SDG would decrease their migration across BBB models. Indeed, application of SDG to monocytes before migration assays resulted in complete reversal of migration to control levels (conditions without CCL2/TNFα) (Fig. 3c). There was no dose-dependent effect of SDG in migration assays.

**Fig. 4:**
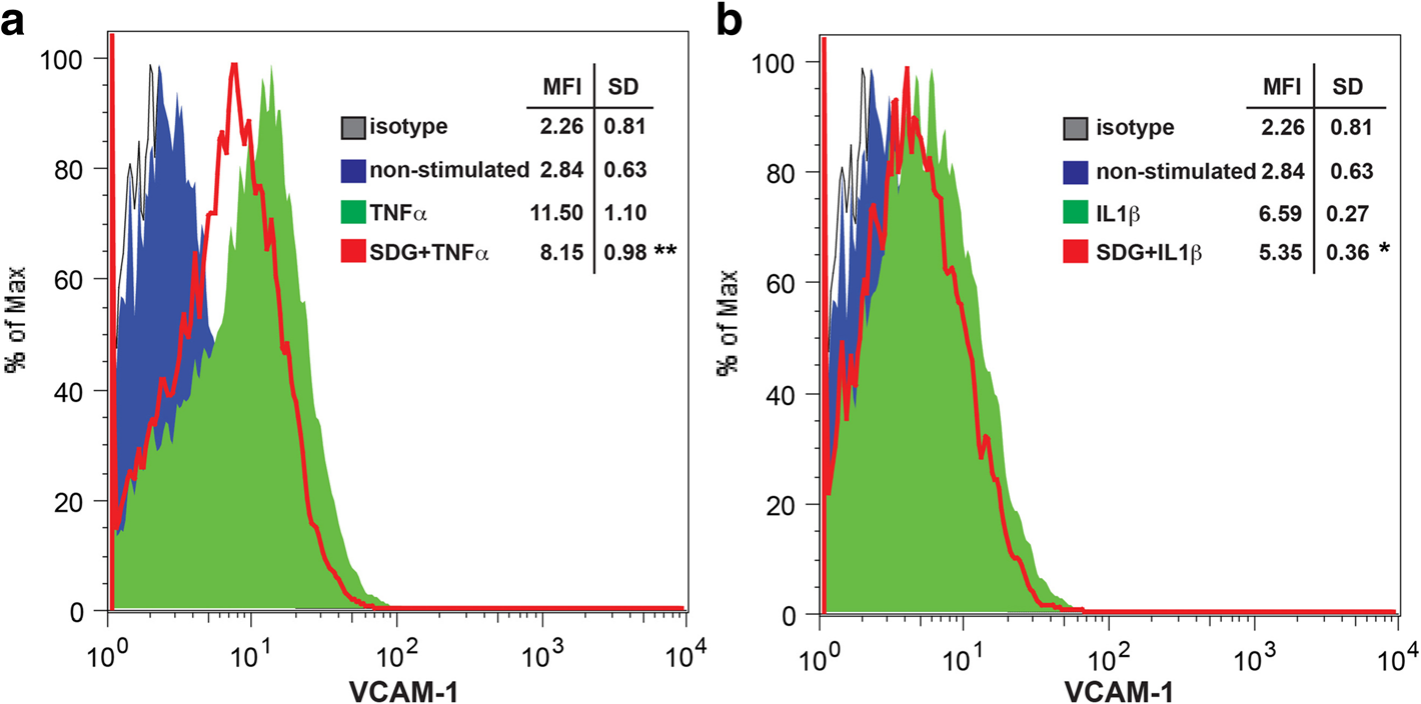
VCAM-1 expression is diminished in BMVEC during inflammation. FACS histograms of VCAM-1 expression in BMVEC pretreated with SDG (50 μM) for 1 h and stimulated with TNFα (100 ng/ml) (**a**) and IL1β (100 ng/ml) (**b**) for 4 h. Ten thousand events were collected. Data are represented as MFI

To investigate the mechanism of diminished adhesion, we tested whether SDG treatment of activated BMVEC diminishes expression of the adhesion molecule VCAM-1. BMVEC stimulation with TNFα resulted in a 4-fold increase of VCAM-1 expression, and SDG reduced it by 35% (Fig. 4a). Similarly, IL1β increased VCAM-1 expression in BMVEC 2.3-fold, and SDG treatment diminished it by 33% (Fig. 4b).

**Fig. 5:**
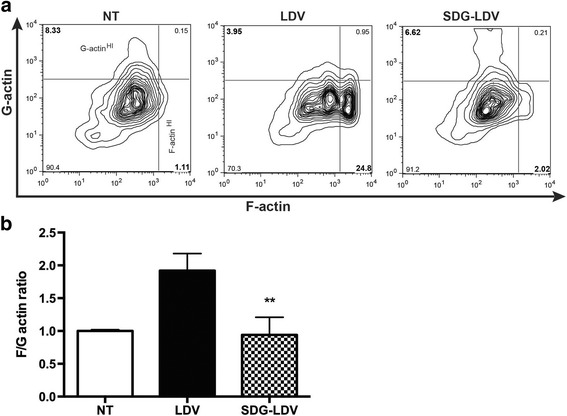
SDG suppresses cytoskeletal re-arrangements in monocytes. FACS analysis of monocytes stimulated by 12 nM LDV peptide (VCAM/ICAM consensus sequence) [28] and treated with SDG (0 or 50 μM) for 4 h. Representative contour plots of FACS reads (**a**). **b** F/G actin ratio in monocytes treated with SDG. Ten thousand events were collected. Ratios of MFIs for F-actin and G-actin were calculated, and the levels of F/G actin in non-stimulated, non-treated cells were assigned a value of 1. Results are shown as the mean ± SD (**p < 0.05 vs. untreated control) from three independent experiments

**Fig. 6:**
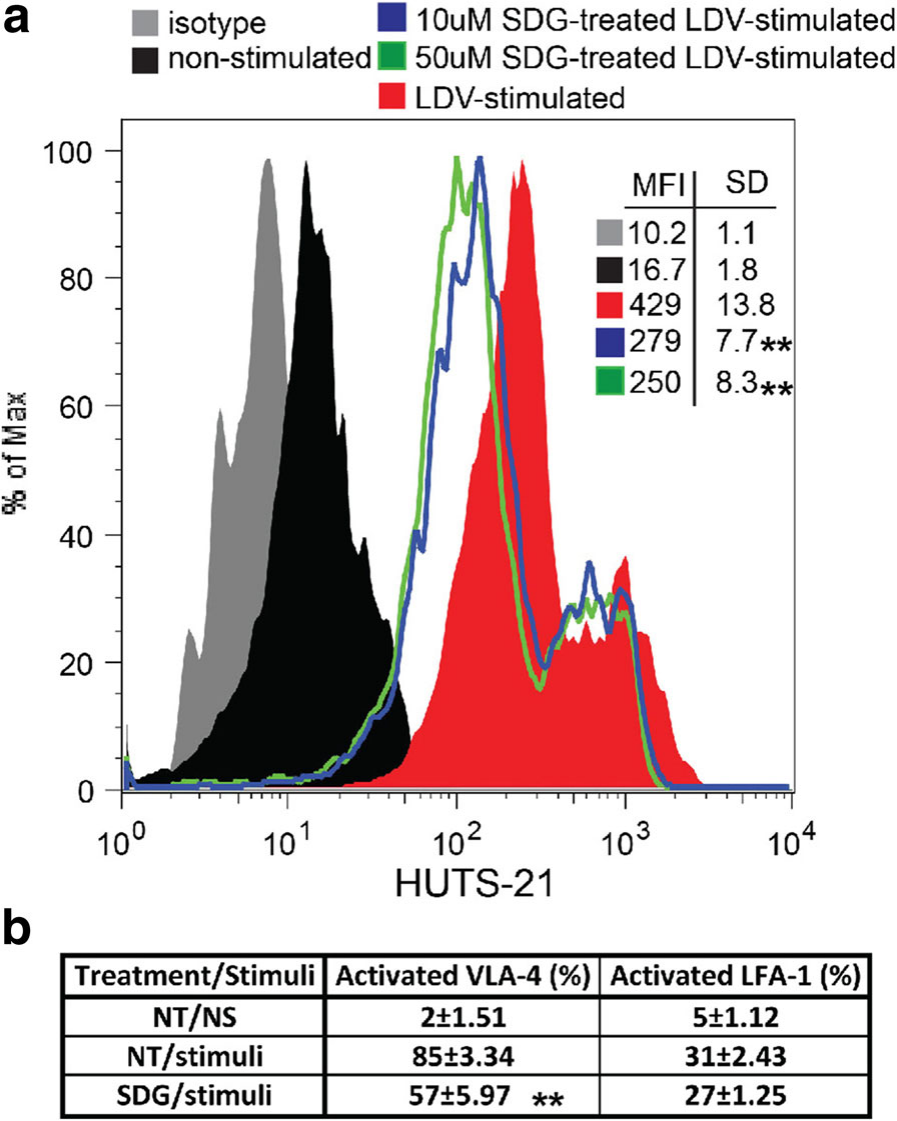
SDG decreases VLA-4 activation in monocytes. FACS analysis of monocytes stimulated by 12 nM LDV peptide (VCAM/ICAM consensus sequence) [28] or with 100 mM PMA for VLA-4 and LFA-1 activation, respectively, and treated with SDG (0, 10, and 50 μM) for 4 h. Representative histograms of VLA-4 activation (with conformational Abs HUTS-21) (**a**). Ten thousand events were collected. Data are presented as MFI. **b** Percent of conformational activation of integrin β1 (LFA-1) and integrin β2 (VLA-4) was calculated as described in “Methods” section. **p < 0.01

Changes in monocyte cytoskeleton are essential for cell migration. To simulate changes in actin seen in inflammation, we stimulated primary human monocytes with LDV (mimicking interactions with adhesion molecules), which led to a 1.95-fold increase in the ratio of fibrilar/globular actin. These cytoskeletal changes in monocytes were completely blocked by SDG (Fig. 5a, b). Monocyte adhesion to activated endothelium is mediated by integrins, such as very late antigen 4 (VLA-4), whose active conformation is stimulated by inside-out or outside-in activation [28]. Expression of the active form of VLA-4 is associated with enhanced monocyte adhesion and migration. To test whether SDG treatment could change expression of the active form of VLA-4, we treated monocytes with the relevant stimulus, LDV peptide, mimicking VLA-4 interactions with VCAM-1, and fibronectin. LDV caused a 26-fold increase in expression of the activated form of VLA-4 and SDG reduced it by 35–42% (Fig. 6). Overall, our results indicate that SDG can attenuate inflammatory changes in BMVEC and monocytes and reduce adhesion/migration of monocytes across BBB models in vitro.

## Discussion

BBB injury is documented in multiple neuroinflammatory disorders (multiple sclerosis, viral encephalitis) and neurodegenerative processes (Alzheimer’s and Parkinson’s diseases) [35, 37, 38]. It plays a role in neuronal dysfunction due to changes in the unique environment of the CNS. Identification of effective anti-inflammatory, non-toxic barrier-protective compounds is therefore of paramount importance. With this goal in mind, we have tested the ability of orally administered SDG to attenuate neuroinflammation at the level of the BBB. Using a model of aseptic meningitis/encephalitis (i.c. TNFα injection) and in vivo microvessel imaging, we have demonstrated a 4-fold increase in leukocyte adhesion and migration across the BBB. SDG administration (100 mg/kg twice daily) diminished adhesion and migration by 50 and 64%, respectively. Prior studies showed attenuation of end-organ injury by SDG in radiation-induced lung [30] and asbestos-provoked acute peritoneal inflammation [7]. Such processes usually are accompanied by leukocyte infiltration, release of pro-inflammatory factors, ROS, and MMPs. Indeed, Pietrofesa and colleagues showed decreases in leukocyte accumulation, cytokine secretion (IL-1ß, IL-6, TNFα, HMGB1, and TGFß1), and cytokine receptors (TNFαR1 and TGFßR1) after SDG administration.

Systemic inflammation accompanied by cytokine release results in leukocyte adhesion to endothelium (including CNS) and enhanced permeability [21, 33]. LPS caused an increase in permeability that was reduced to control levels by SDG feeding, indicating barrier-protective effects of the compound (Fig 2).

In vitro experiments supported our in vivo observations; pretreatment of BMVEC with different concentrations of SDG (0, 1, 2, 5, 10, or 50 μM) diminished primary monocyte adhesion to and migration across BMVEC monolayers (model of BBB) in a dose-response manner. Significant effects on leukocyte adhesion were achieved only at 10 or 50 μM, whether BMVEC or monocytes were SDG-treated (Fig. 3a). Interestingly, the ability of monocytes to migrate through BMVEC monolayers was significantly affected only at 10 μM when BMVEC were SDG-treated, and at 2, 5, and 10 μM when monocytes were SDG-treated (Fig. 3b). Since 10 μM showed a more consistent outcome (*p* value of less than 0.001), the rest of the experiments were performed at this concentration. To explore the mechanisms of the anti-inflammatory SDG effects, we analyzed expression of VCAM-1 after stimulation with TNFα or IL-1β. Enhanced expression of VCAM-1 (2.3- and 4-fold, respectively) was decreased by 33–35% by SDG suggesting that VCAM reduction could be one of the factors in attenuated adhesion and migration after SDG treatment (Fig. 4). Protective effects of SDG on the microvasculature have been shown in a cardiac infarct model, including diminished infarct volume, enhanced expression of eNOS, vascular endothelial growth factor, and hemeoxygenase-1 [39]. Our findings extend the protective effects of SDG to the BBB, which has unique permeability properties and distinct contributions to an array of neuroinflammatory diseases including viral encephalitides and neurodegerative diseases.

Protective properties of SDG could also be attributed to anti-inflammatory effects on leukocytes in addition to brain endothelium. We have previously demonstrated that anti-inflammatory compounds (like PARP or GSK3β inhibitors [28, 29]) can lead to conformational changes of active integrin and/or total integrin expression. Stimulation with the LDV peptide, a VCAM/ICAM consensus sequence mimicking monocyte integrin interactions with these adhesion molecules, increased expression of active VLA-4 26-fold while treatment with SDG attenuated active integrin β1 expression by 35–42% (Fig. 6). Conformational changes in integrins following leukocyte interactions reveal the VCAM-1 binding site [40], enabling both tethering and rolling of leukocytes [41]. Memory T cells that permanently display activation/ligand-induced epitopes on β1 integrins, usually have significantly higher rates of attachment to VCAM-1 expressing cells as compared to other T cell subsets without active epitope expression [28, 42]. Previously, we [25, 28] and others [43] demonstrated an association between conformational VLA-4 activation and Rac1 pathways (employing inhibitors of GSK3β, PARP, or Rac-1), resulting in attenuated expression of the active VLA-4 form and decreased monocyte adhesion/migration across monolayers of BMVEC [25, 28].

Previous studies suggested an association between active VLA-4 form with specific cytoskeletal changes [29] and diminished lamellipodia formation [44]. We explored the possibility that SDG would prevent cytoskeletal changes present in leukocytes with a ‘migration’ phenotype. Importantly, human monocyte stimulation with LDV led to a 1.95-fold increase in fibrillary/globular actin ratio, a typical change seen in actin during inflammation [45]; these skeletal changes were completely blocked by SDG (Fig. 5).

SDG has been shown to mitigate pathology in several disease models where oxidative stress and inflammation play a prominent role, including atherosclerosis, diabetes, lung disease from radiation exposure, liver and kidney diseases [2–6, 9, 10, 18]. The effects of SDG on lung macrophages have been explored and suggest that SDG exerts its protective effects via induction of the endogenous antioxidant response, dampening inflammatory signaling, and free radical scavenging; however, its role in endothelial cells has not been examined. This study suggests that SDG directly inhibits BBB interactions with inflammatory cells while also reducing the inflammatory state of leukocytes. Although more work is needed to determine the mechanism by which SDG mediates these effects, the potential ability of SDG to exert a multi-functional response to reduce oxidative stress, inflammation, and BBB permeability make it an exciting potential therapeutic for neuroinflammatory diseases. To this end, numerous studies have shown that SDG formulations are safe and well tolerated in animals and importantly in humans with diverse pathologies [4, 10, 12, 13]. Together, our findings indicate that SDG may be considered as a therapeutic agent for neuroinflammatory diseases.

## Conclusion

This study indicates that SDG directly inhibits BBB interactions with inflammatory cells while also reducing the inflammatory state of leukocytes. Although further effort is desired to define the machinery by which SDG facilitates these effects, the ability of SDG to elicit a multi-functional response to diminish inflammation, oxidative stress, and BBB permeability make it an exciting potential therapeutic for neuroinflammatory diseases. SDG may serve as an anti-inflammatory and barrier-protective agent in neuroinflammation.

## Abbreviations

BBB: Blood brain barrier
BMVEC: Brain microvascular endothelial cells
EAR: Endogenous antioxidant response
FACS: Flow cytometry
IL1β: Interleukin type 1 beta
LPS: Lipopolysaccharide
MCP1: Monocyte chemotactic protein type 1
MFI: Mean fluorescence intensity
SDG: Secoisolariciresinol diglucoside
SEM: Standard error of the mean
TNFα: Tumor necrosis factor alpha
VCAM-1: Vascular cell adhesion molecule 1
VLA-4: Very late antigen 4
